# A molecular census of early‐life stage scleractinian corals in shallow and mesophotic zones

**DOI:** 10.1002/ece3.8122

**Published:** 2021-10-19

**Authors:** Derek Soto, Stéphane De Palmas, Ming‐Jay Ho, Vianney Denis, Chaolun Allen Chen

**Affiliations:** ^1^ Biodiversity Program Taiwan International Graduate Program Academia Sinica and National Taiwan Normal University Taipei Taiwan; ^2^ Biodiversity Research Center Academia Sinica Taipei Taiwan; ^3^ Department of Life Science National Taiwan Normal University Taipei Taiwan; ^4^ Green Island Marine Research Station Academia Sinica Ludao, Taitung County Taiwan; ^5^ Institute of Oceanography National Taiwan University Taipei Taiwan; ^6^ Department of Life Science Tung Hai University Taichung Taiwan

**Keywords:** barcoding, diversity, recruitment, refuge, settlement, Taiwan

## Abstract

The decline of coral reefs has fueled interest in determining whether mesophotic reefs can shield against disturbances and help replenish deteriorated shallower reefs. In this study, we characterized spatial (horizontal and vertical) and seasonal patterns of diversity in coral recruits from Dabaisha and Guiwan reefs at Ludao, Taiwan. Concrete blocks supporting terra‐cotta tiles were placed at shallow (15m) and mesophotic (40m) depths, during 2016–2018. Half of the tiles were retrieved and replaced biannually over three 6‐month surveys (short‐term); the remainder retrieved at the end of the 18‐month (long‐term) survey. 451 recruits were located using fluorescent censusing and identified by DNA barcoding. Barcoding the mitochondrial cytochrome oxidase I (*COI*) gene resulted in 17 molecular operational taxonomic units (MOTUs). To obtain taxonomic resolution to the generic level, *Pocillopora* were phylotyped using the mitochondrial open reading frame (*ORF*), resolving eight MOTUs. *Acropora*, *Isopora*, and *Montipora* recruits were identified by the nuclear *PaxC* intron, yielding ten MOTUs. Overall, 35 MOTUs were generated and were comprised primarily of *Pocillopora* and, in fewer numbers, *Acropora*, *Isopora*, *Pavona*, *Montipora*, *Stylophora*, among others. 40% of MOTUs recruited solely within mesophotic reefs while 20% were shared by both depth zones. MOTUs recruiting across a broad depth distribution appear consistent with the hypothesis of mesophotic reefs acting as a refuge for shallow‐water coral reefs. In contrast, *Acropora* and *Isopora* MOTUs were structured across depth zones representing an exception to this hypothesis. This research provides an imperative assessment of coral recruitment in understudied mesophotic reefs and imparts insight into the refuge hypothesis.

## Graphical abstract

We characterized spatial (horizontal and vertical) and seasonal patterns of diversity in coral recruits at shallow and mesophotic reefs in Ludao, Taiwan, using DNA barcoding of the *COI*, *PAXC*, and *ORF* regions. Thirty‐five MOTUs were generated and were comprised primarily of *Pocillopora* and, in fewer numbers, *Acropora*, *Isopora*, *Pavona*, *Montipora*, *Stylophora*, among others. MOTUs recruiting across a broad depth distribution appear consistent with the hypothesis of mesophotic reefs acting as a refuge for shallow‐water coral reefs.

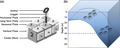

## 1 BACKGROUND

Climate change and other human disturbances have propelled an ongoing decline in coral reefs worldwide, and the outlook of reefs remains bleak (Hoegh‐Guldberg et al., 2018; Hughes et al., 2017; Van Hooidonk et al., 2016). Mesophotic coral ecosystems (MCEs)—coral reefs between 30 and 150m—may shelter against disturbances which affect shallower reefs (< 30m) and may propagate larvae to recruit on impacted shallow reef ecosystems (Baird et al., 2018; Bongaerts et al., 2010; Bongaerts & Smith, 2019). Reproduction in corals is induced by environmental cues, such as the synergic properties of increasing light and temperature (reviewed in Harrison (2011)). Because these signals attenuate with depth (Kahng et al., 2019), it is plausible that patterns of recruitment in mesophotic corals may contrast from corals in shallow reefs (Prasetia et al., 2017; Shlesinger et al., 2018). Most mesophotic corals studied to date (9 of 11) have exhibited reduced fecundity and gamete size compared to shallow‐water conspecifics, resulting in diminished larval supply (Eyal‐Shaham et al., 2016; Holstein et al., 2016; Prasetia et al., 2016, 2017; Shlesinger et al., 2018; Smith et al., 2016). Studies examining genetic connectivity in *Montastraea cavernosa* (Brazeau et al., 2013; Eckert et al., 2019; Serrano et al., 2014), *Porites astreoides* (Serrano et al., 2016), and *Agaricia fragilis* (Bongaerts et al., 2017) reveal population structures partitioned across shallow and deep zones, indicating that vertical genetic exchange is restricted. In contrast, similar analyses in *Stephanocoenia intersepta* (Bongaerts et al., 2017), *Agaricia lamarcki* (Hammerman et al., 2018), and *Pocillopora verrucosa* (de Palmas, 2020) support well‐mixed cohorts. Interpreted together, these results suggest that larval exchange across vertical gradients is likely location‐ and/or species‐specific.

Artificial units of recruitment (AURs) have been employed by researchers to assess coral recruitment for more than 100 years (Mundy, 2000), and their application to collect and study recruits has seen widespread use (Field et al., 2007; Hill & Wilkinson, 2004). Yet, the availability of research targeting recruitment decreases inversely with depth, constrained by the technical challenges associated with working beyond the recreational scuba depth limit. Historically, few studies have examined patterns of recruitment at depths beyond 30m (Bak & Engel, 1979; Birkeland, 1977; Birkeland et al., 1981; Hughes & Tanner, 2000; Rogers et al., 1984; Vermeij et al., 2011). As of 2017, only 1% of research targeting MCEs had examined recruitment (Turner et al., 2017); however, improved availability of diving and ROV technology and the development of new methods to study MCEs are accelerating this research. For example, Turner et al. (2018), innovated an approach to examine patterns of recruitment at 40m depth in Western Australia which foregoes diving. Also, Kramer et al. (2019) used technical diving to describe recruitment dynamics at 50m depth in the Red Sea. Albelda et al. (2020) used conventional scuba to compare juvenile and adult assemblages down to 40m depth in the Philippines. Despite recent progress, our knowledge of recruitment in MCEs remains limited and many geographic areas have never been studied, emphasizing the need for more research.

Another challenging aspect of studying the early‐life history of corals lies in locating small recruits and identifying them based on few useful morphological characters (Green & Edmunds, 2011). During settlement, spats metamorphose into their benthic life stage and begin accretion of the corallite matrix (Gilis et al., 2014), on which discrimination of microstructural characters is based (Budd & Stolarski, 2011). Fluorescence has proven useful for locating and identifying corals during this developmental stage (Baird et al., 2006; Eyal et al., 2015; Hsu et al., 2014; Roth et al., 2013); nevertheless, identification of recruits based on morphological traits is mainly limited to the family level (Babcock et al., 2003; Green & Edmunds, 2011; Nozawa et al., 2013). This limitation is particularly important in locations where diversity is high and traits converge between confamiliar taxa (Baird & Babcock, 2000), such as the Indo‐Pacific. An inability to identify recruits complicates ecological assessments and hinders our ability to deduce the outlook of threatened coral communities (O’Cain et al., 2019), particularly when confamiliar species fulfill divergent functional roles within an ecosystem (Denis et al., 2017). Therefore, leveraging molecular typing toward improving the taxonomic resolution of early‐life stage communities (Hsu et al., 2014; O’Cain et al., 2019; Shearer & Coffroth, 2006) can yield new insights.

This study details a census and comparison of coral recruitment in shallow and mesophotic reef communities at Ludao, an island off southwestern Taiwan. Shallow and mesophotic communities at Ludao have been shown to possess distinctive communities, despite similarities in coral assemblages (Lin & Denis, 2019). In the past, assessments of recruitment in Taiwan were conducted above 15m (Edmunds et al., 2014; Ho & Dai, 2014; Nozawa et al., 2013; Soong et al., 2003) and all have identified recruits morphologically, except Hsu et al. (2014) which identified recruits by barcoding. Here, we employ technical scuba diving and a sampling design in which tiles are fixed to blocks to survey recruitment within shallow and mesophotic zones. We censused recruits with the aid of fluorescent and white light and then barcoded their DNA using three molecular markers to identify recruits and generate molecular operational taxonomic units (MOTUs).

## 2 MATERIALS AND METHODS

Recruitment was surveyed off the coast of Ludao Island, situated 33 km southeast of Taiwan, during April 2017–October 2018. Two shallow (14–16 m; hereafter 15m) and two mesophotic sites (38–42 m; hereafter 40m) were selected according to habitat composition, favorable wind, wave and current conditions, and accessibility: Guiwan 15m (22°36'35.57" N, 121°28'57.10" E), Guiwan 40m (22°38’24.59" N, 121°28’51.89" E), Dabaisha 15m (22°38'11.86" N, 121°29'31.24" E), and Dabaisha 40m (22°38'09.08" N, 121°29'21.44" E). Shallow reefs at Guiwan and Dabaisha consist of fringing reef structures which are subjected to high‐energy hydrodynamic transport (Lau et al., 2015). These benthic communities are characterized by arborescent, bushy, and tabular hard corals, clustered octocorals, and encrusting actinarians (Lin & Denis, 2019). MCEs at Guiwan and Dabaisha exhibit limited hard substrates interspersed with sediment and rubble (Denis et al., 2019). Communities at mesophotic sites are denoted by unattached hard corals, bushy and encrusting octocorals, massive sponges, encrusting ascidians, filamentous cyanobacterians, and bushy hydrozoans (Lin & Denis, 2019).

**FIGURE 1:**
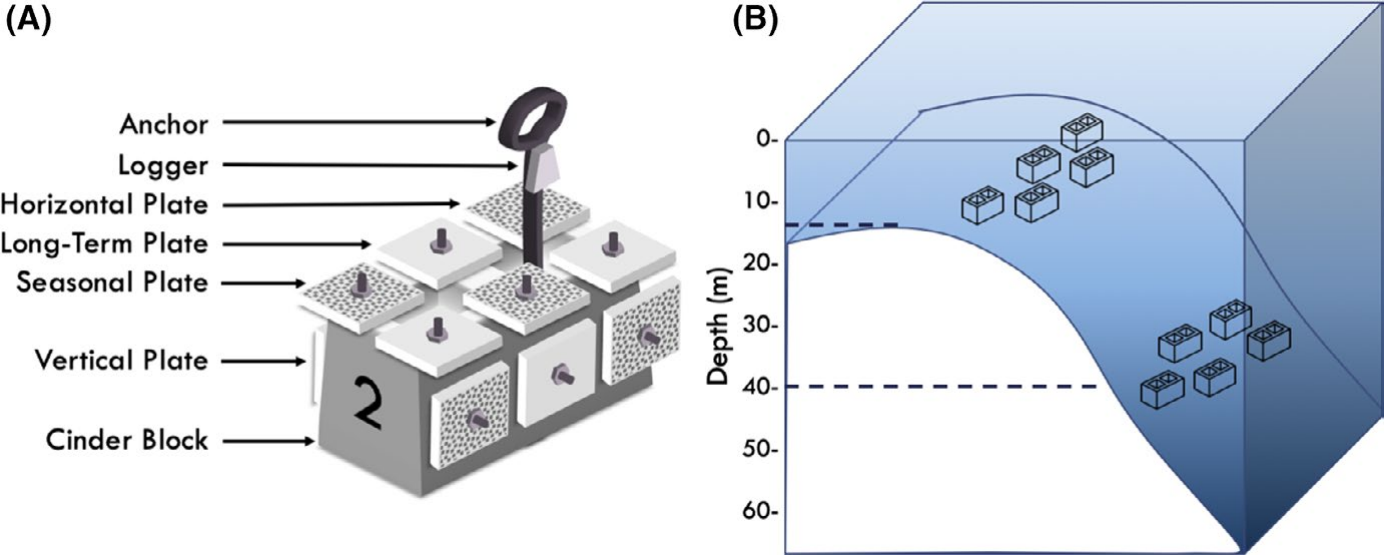
a. Experimental design. Artificial unit of recruitment (AUR): a concrete block filled with cement for ballast and anchored to the seafloor with a rebar. Each block supports 12 plates: 6 in vertical orientation and 6 in horizontal orientation. Plate shading identifies seasonal and long‐term plate placement. b. Schematic of blocks on reef (not to scale)

Artificial units of recruitment (AURs) were constructed from concrete blocks drilled to accept 12 stainless steel concrete sleeve anchors: six on the superior surface and three on each opposing lateral surface (Figure 1a). A labeled terra‐cotta tile measuring 12.5 × 12.5 × 1 cm (total surface area 362.5 cm^2^/tile) was fastened to each sleeve using a stainless steel washer and nut, resulting in an arrangement of 12 plates per block. Plates were fixed at a height of approximately 7 cm from the face of the block. Six seasonal and six long‐term tiles were distributed between the superior and lateral faces of the block using a Latin square design, producing an arrangement of three vertical long‐term tiles, three vertical seasonal tiles, three horizontal long‐term tiles, and three horizontal seasonal tiles. AURs were deployed during 4–8 April 2017, a few days prior to the mass coral spawning date, expected between 1 and 11 days after the full moon (full moon: 11 April 2017). In total, 20 AURs were deployed during the survey: five AURs were deployed at shallow, and upper mesophotic zones at Guiwan and Dabaisha (Figure 1b), respectively. Seasonal tiles were deployed and retrieved at three 6‐month intervals [Season 1 (S1): April–October 2017, Season 2 (S2): October 2017–April 2018, Season 3 (S3): April–October 2018]. Long‐term (LT) tiles remained in place throughout and were retrieved at the end of the study period (April 2017–October 2018). Seasonal sampling consisted of 30 tiles per depth, per site, per season. Retrieved tiles were organized in numbered resealable bags and maintained in bins filled with circulating seawater to preserve them until processing. Coral recruits, defined as individuals that successfully settled and survived until the time of collection, were detected by visually inspecting tiles under fluorescent light, according to the methodology described in Hsu et al. (2014). Spats were located and photographed under fluorescent blue light (Night Sea BB67), filtered through long‐pass barrier (>500 nm wavelength) filter glasses (Night Sea VG1). An additional survey under white light verified that weakly fluorescent and nonfluorescent spats were collected. All tile surfaces: top, bottom, and four sides, were inspected for recruits. Spats were removed by scraping with a small chisel and preserved in tagged vials containing 99% ethanol. Tiles and remaining assemblages were tagged, bleached, and dried for storage as vouchers.

DNA was extracted using DNEasy Blood and Tissue kits (Qiagen) following the manufacturer's instructions but with slight modifications to increase DNA yield. Samples were incubated overnight in lysis buffer/proteinase K solution, and an additional centrifugation was performed (16,000g for 3 min) before DNA elution in order to completely dry the filter column. DNA concentration and purity were verified using a NanoDrop Spectrophotometer (Thermo Scientific). DNA was serially diluted to achieve a concentration approximating 10 ng/ml. PCR was amplified using 15 μl Taq 2X Master Mix (Amplicon) diluted to achieve a 30 μl total reaction volume. Coral spat DNA was phylotyped using primers which amplify the mitochondrial cytochrome oxidase subunit I (*COI*) region, commonly used to differentiate metazoan invertebrates (Folmer et al., 1994). *COI* primers: LCO1490: 5′‐GGT CAA CAA ATC ATA AAG ATA TTG G‐3′ and HCO2198: 5′‐TAA ACT TCA GGG TGA CCA AAA AAT CA‐3′. Thermal cycling for *COI* entailed an initial heating at 95°C for 3 min followed by 30 cycles at 95°C for 60 s, 45°C for 60s, and 72°C for 90s, and a final elongation step of 7 min at 72°C. Spats identified as Pocilloporidae were further resolved using the mitochondrial open reading frame (*ORF*) region as described in Chen et al. (2008). *ORF* primers: FATP6.1:5′‐TTT GGG SAT TCG TTT AGC AG‐3′ and RORF: 5′‐SCC AAT ATG TTA AAC ASC ATG TCA‐3′. Thermal cycling for *ORF* comprised an initial heating at 94°C for 3 min followed by 40 cycles at 94°C for 30 s, 53°C for 30 s, and 72°C for 90 s, and a final elongation step of 7 min at 72°C. Spats placed by *COI* within the Acroporidae were subjected to additional PCR using the nuclear *PaxC* 46/47 intron (van Oppen et al., 2011). *PaxC* primers: *PaxC*_intron‐FP1: 5′‐TCC AGA GCA GTT AGA GAT GCT GG‐3′ and *PaxC*_intron‐RP1: 5′‐GGC GAT TTG AGA ACC AAA CCT GTA‐3′. PCR protocol for *PaxC* consisted of a 95°C denaturation step for 3 min, followed by 5 cycles of 30 s at 94°C, 30 s at 50°C, 1 min at 72°C, followed by 26 cycles with an annealing temperature of 56°C, and a final elongation of 7 min at 72°C. Reactions were verified using 2% agar gel electrophoresis and fluorescent gel staining prior to outsourcing for Sanger sequencing.

Raw forward and reverse sequences were screened by querying the NCBI BLAST database (Megablast) (Altschul et al., 1990), and sequences not matching scleractinian corals were excluded from further analysis. Using UGENE v.1.31 (Okonechnikov et al., 2012), DNA sequences were assembled, and consensus sequences were created from forward and reverse reads and aligned using the MUSCLE algorithm (Edgar, 2004). *COI* sequences were compared to GenBank sequences of the same marker matching the search terms “scleractinia” (Benson et al., 2005). PHYLIP neighbor‐joining trees consisting of sample and reference sequences were generated under the F84 distance matrix model in UGENE (Felsenstein, 2004). Molecular operational taxonomic units were generated using a furthest neighbor clustering algorithm defining MOTUs on 5% dissimilarity. Sequences identified with *COI* as Pocilloporidae underwent additional phylotyping of the *ORF* region and those identified as Acroporidae were typed with *PaxC*. *PaxC* and *ORF* sequences were aligned with GenBank reference sequences which matched the search terms “acroporidae” and “pocilloporidae,” respectively. MOTUs for these markers were assigned following the same protocol used for the *COI* marker. Finalized phylogenetic trees were rendered using the interactive Tree of Life (iTOL) (Letunic & Bork, 2019). Pearson's chi‐squared tests were performed using the “stats” package in R version 3.4.2 (R Core Team, 2013).

## 3 RESULTS

We collected 518 coral‐like spats and identified 451 coral spats through the combined barcoding of *COI*, *ORF*, and *PaxC*. Recruitment averaged 19.1 ± 80.0 recruits/m^2^ throughout the study period. 55.5% of spats originated from shallow AURs and 44.5% from mesophotic AURs. 65.3% of spats recruited on horizontally oriented tiles and 34.7% recruited on vertically oriented tiles. Sixty‐seven samples were excluded from our analysis, representing 7.7% of the overall collection: 37 specimens phylotyped as nonscleractinian invertebrates, 22 spats for which no PCR product was obtained, or which did not return any matches in BLAST database searches, 5 pocilloporid samples which did not amplify *ORF*, and 3 acroporid samples which did not amplify *PaxC*. Recruitment was more abundant during S1 (150 recruits) and S3 (126 recruits), largely exceeding S2 (24 recruits).

**FIGURE 2:**
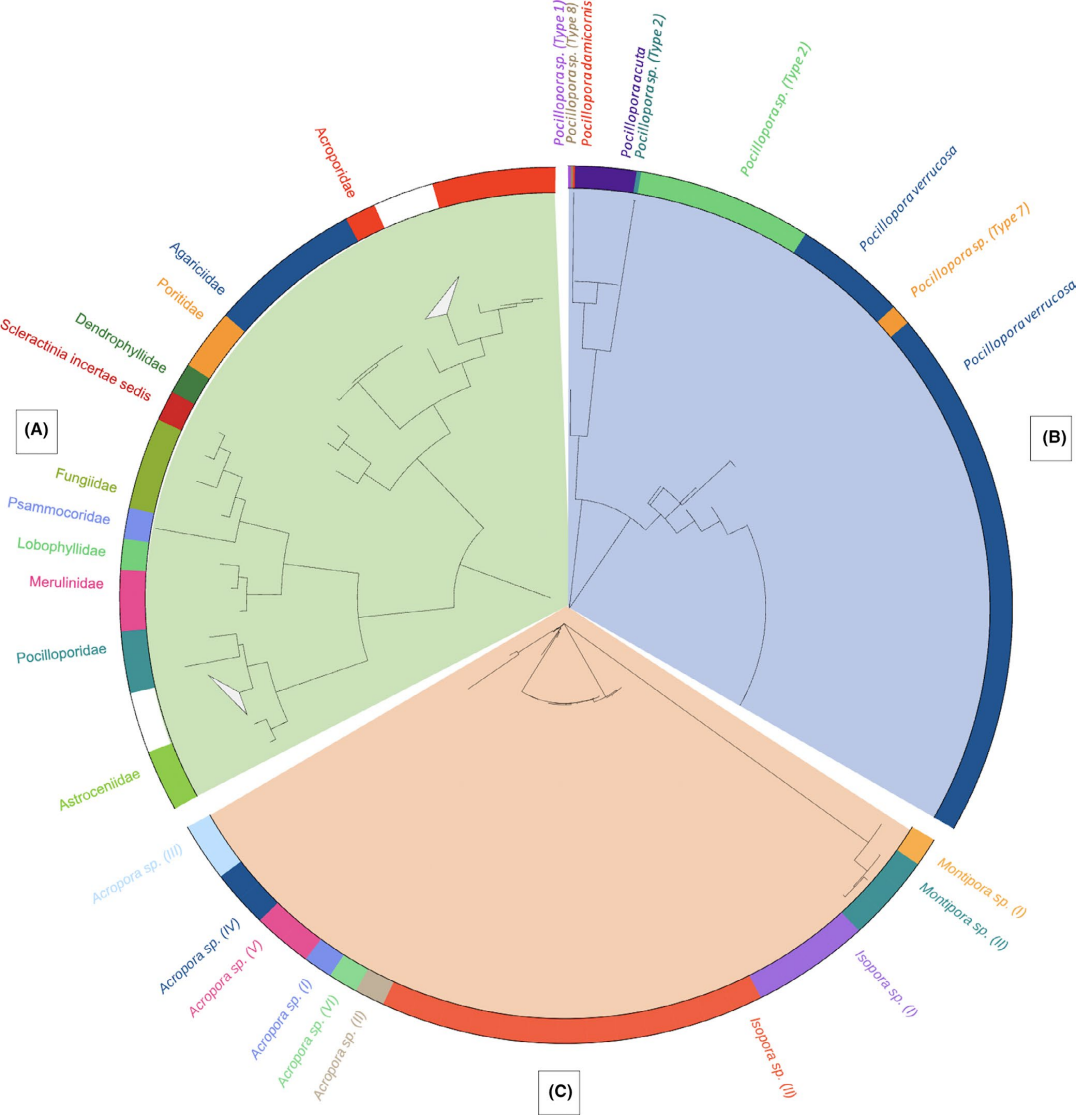
Neighbor‐joining tree of a. *COI*, b. *ORF*, and c. *PaxC* barcoded recruits. Collapsed branches in a. represent *Acropora* and *Pocillopora*, respectively

Phylogenetic analysis of *COI* sequences (Figure 2) distinguished complex and robust clades. We identified 348 Pocilloporidae: 346 *Pocillopora* spp. and two *Stylophora pistillata*. *Thirty‐one* recruits were classified as Acroporidae: 17 *Isopora* spp., nine *Acropora* spp., four *Montipora* spp., and a single *Astreopora* sp. We also collected five Agariicid recruits: four *Pavona* sp. (I) and another *Pavona sp*. of a different haplotype (II). Also, two Astrocoeniidae haplotypes: one *Stylocoeniella* sp. (I) and one *Stylocoeniella* sp. (II) and three Fungiidae genera were recovered: *Lobactis* sp., *Cycloseris* sp., and *Sinuorota* sp. Poritidae were represented by two individual haplotypes: *Porites* sp. (I) and *Porites* sp. (II) Single individuals represented the Dendrophyllidae, Merulinidae, Psammocoridae, and Lobophyllidae families, respectively: *Dipsastraea* sp., *Goniastrea* sp., *Psammocora* sp., and *Lobophyllia* sp.. *Leptastreidae* sp. is temporarily unclassified (Scleractinia incertae sedis). A single azooxanthellate coral, *Dendrophyllia* sp., was also identified.

Using the *ORF* marker, we barcoded 399 samples comprising 346 recruits classified with *COI* as *Pocillopora*, and an additional 53 samples which were unidentifiable with *COI* but which amplified with the *ORF* marker. Additionally, the identities of two recruits typed with *COI* as *Stylophora pistillata* were verified. Phylogenetic analysis of the *ORF* marker (Figure 2b) identified eight *Pocillopora* MOTUs. Overall, the most abundant MOTU retrieved in this study was *Pocillopora verrucosa*, comprising 282 recruits, and representing 62.5% of all identified recruits and 70% of all pocilloporids. We also identified 76 *Pocillopora grandis*, 27 *Pocillopora acuta*, nine *Pocillopora* sp. (Type 7), two *Pocillopora* sp. (Type 2), and three unique taxa: *Pocillopora damicornis*, *Pocillopora* sp. (Type 1), and *Pocillopora* sp. (Type 8).

Thirty recruits typed with *COI* as *Acropora*, *Isopora*, or *Montipora* were resolved further with the *PaxC* marker and resolved into ten MOTUs (Figure 2c). *Isopora* sp. (I), likely *I. palifera*, was the most abundant taxon (*n* = 14), comprising almost half of acroporids found. *PaxC* genotyping also confirmed three *Isopora* sp. (II), four *Montipora* sp. (I), two *Acropora* sp. (III), two *Acropora* sp. (IV), two *Acropora* sp. (V), and four unique MOTUs: *Acropora* sp. (I), *Acropora* sp. (VI), *Acropora* sp. (II), and *Montipora* sp. (II).

**FIGURE 3:**
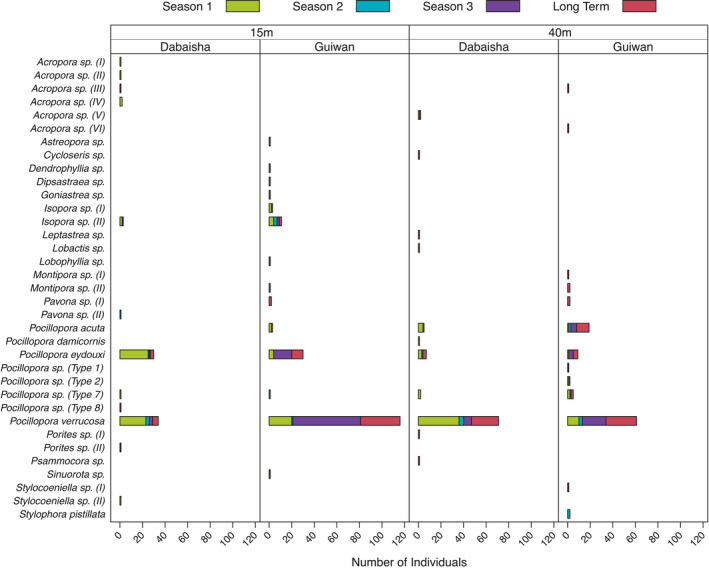
Summary of MOTUs generated from barcoded COI, ORF, and PaxC markers, sorted by seasons, sites, and depth zones, in alphabetical order

Overall, we identified 35 MOTUs from coral recruits barcoded *COI*, *ORF*, and *PaxC* markers, (Figure 3): 17 MOTUs derived from the *COI* marker, eight MOTUs were obtained from *ORF* barcoding, and ten MOTUs from *PaxC*. 15 MOTUs recruited only within the shallow zone, while 13 were found only in the mesophotic zone and seven MOTUs recruited at both depths (Figure 3). Thirteen MOTUs recruited during S1, 9 MOTUs during S3, and 8 MOTUs in S2. Seasonality (χ^2^ = 152.6, *p* < .005), site (χ^2^ = 50.8, *p* = .03), and depth (χ^2^ = 88.6, *p* < .005) significantly impacted the distribution of MOTUs in this study. When acroporids were excluded, depth (χ^2^ = 65.4, *p* < .001), and season (χ^2^ = 123.3, *p* < .001) remained significant but sites did not (χ^2^ = 34.2, *p* = .079).

## 4 DISCUSSION

We collected and identified 451 recruits, comprising 35 MOTUs and 13 coral families. Many recruits—the smallest of which possessed a diameter of 0.7mm—were too small to reliably identify morphological characters which would enable identification beyond the family tier. Unidentified samples composed 4% of our overall collection and reflect a marked improvement compared to a similar study using a high‐salt DNA extraction method (9.7%)(Hsu et al., 2014). At least 328 scleractinian coral species inhabit the reefs of Taiwan and 8 MOTUs identified in this study have been found in previous diversity surveys (Dai & Horng, 2009a, 2009b; Denis et al., 2015, 2019; de Palmas et al., 2018; Huang et al., 2015). Past synonymization of “*Pocillopora damicornis*‐like” coral morphologies (Poquita‐Du et al., 2017; Schmidt‐Roach et al., 2014) has obscured the diversity of *Pocillopora acuta* in previous surveys of Taiwan, explaining its absence in species catalogs. Nevertheless, recent studies have begun addressing this deficiency. Their presence could indicate long‐distance larval transport and/or undocumented diversity in Taiwan; however, more intensive surveys are required to verify this inference. Ninety‐six scleractinian coral species have been found to inhabit Taiwanese mesophotic reefs to date (Denis et al., 2019), of which our survey found three: *P*. *damicornis*, *P. eydouxi*, and *P. verrucosa*. Because these species can also be found on shallow reefs, vertical larval transport seems plausible. Denis et al. (2019) also list ten coral species known to inhabit the mesophotic zone exclusively, yet none were identified in our survey.

Pocilloporidae were abundantly represented throughout our study, comprising 401 individuals and nine MOTUs including the *Pocillopora* and *Stylophora* genera. This pattern is consistent with other Taiwanese surveys where Pocilloporidae were the most proliferous group (Edmunds et al., 2014; Ho & Dai, 2014; Hsu et al., 2014; Kuo & Soong, 2010; Soong et al., 2003). The *Pocillopora* genus comprised 88% of overall recruitment but contributed only 27% of overall MOTUs. *Pocillopora* is a genus notoriously difficult to identify solely based on morphology and its diversity might be underestimated when forgoing genetic identification (de Palmas et al., 2018; Soto et al., 2018). For example, the synonymization of “*Pocillopora damicornis*‐like” coral morphologies (Poquita‐Du et al., 2017; Schmidt‐Roach et al., 2014) has obscured the diversity of *Pocillopora acuta* in previous surveys of Taiwan. This issue which has only been addressed recently (Mayfield et al., 2018) and may explain the absence of *P. acuta* in older species catalogs but its detection by genetic assays. In Ludao, *P. verrucosa* exhibits a wide bathymetric distribution and constitutes one of the dominant corals structuring shallow and mesophotic seascapes. In addition, *P. verrucosa* is a functionally competitive species, capable of sustained recruitment (Kayal et al., 2018), explaining its position as the most copious recruiter here.

Acroporidae was the only taxonomic group to exhibit clear bathymetric structuration, and only one MOTU, *Acropora* sp. (III)*,* was present at both depth zones. At least 90 Acroporidae species are currently documented in Taiwan and this family yielded relatively high richness (11 MOTUs), but low abundance (*n* = 31). Some acroporids are shallow‐dwelling and rapid‐growing species and exhibit competitive life‐history traits (Darling et al., 2012). In a study of northern Taiwan, Acroporidae were the most abundant family in long‐term surveys and formed the largest spats, suggesting superior survivorship compared to other families (Ho & Dai, 2014). Another study in Ludao found vertically structured distribution in acroporid recruitment, recruiting abundantly at 5m, but not at 15m, where pocilloporid and poritid recruits dominated instead (Nozawa et al., 2013). It is therefore plausible that greater diversity of Acroporidae may have been captured by surveying shallower. In addition, acroporids are vulnerable to physical disturbances such as storms (Madin, 2005), which have severely impacted local populations (Chen & Dai, 2004; Kuo et al., 2011, 2012). It is possible that low acroporid coral cover locally (Ribas‐Deulofeu et al., 2016) may have led to scarce recruitment in our study, as recruitment in this group is subject to density‐dependent effects (Kayal et al., 2015, 2018).

Poritidae were significant recruiters in previous surveys around Taiwan (Edmunds et al., 2014; Ho & Dai, 2014; Hsu et al., 2014; Kuo & Soong, 2010; Nozawa et al., 2013), but were rare in ours. Massive Indo‐Pacific poritids and many merulinids are associated with a stress‐resistant life‐history strategy characterized by slow growth, long generation times, and sustained recruitment (Darling et al., 2012). However, these types of corals can survive long periods in the absence of recruitment (Hughes & Tanner, 2000); therefore, low recruitment in those taxa, while unusual, is not entirely uncharacteristic of this type of life history.

The potential for MCEs to reseed shallow reefs may apply to species with wide depth distributions which inhabit both depths (Bongaerts & Smith, 2019). Of 20 MOTUs found recruiting within the mesophotic zone, 14 (40%) recruited solely within deeper reefs and only in low abundances. Only seven MOTUs (20%) recruited at both depth zones and four of these were *Pocillopora* (*Pocillopora* sp. (Type 7), *P. acuta*, *P. eydouxi*, *P.verrucosa)*. Research supports genetic connectivity in *Pocillopora verrucosa* across a shallow–mesophotic gradient (de Palmas, 2020), indicating compatibility with the deep reef refuge hypothesis (Bongaerts et al., 2010). Therefore, we hypothesize that the other *Pocillopora* MOTUs, which recruit at both depth zones, may be genetically connected across this depth gradient as well; however, additional research aimed at discriminating patterns of population genetics is required. *Pavona* sp. (I) and *Montipora* sp. (II) also recruited at both depths; however, sparse recruitment limits the interpretation of these patterns. Based on the evidence presented here, the potential for refuge in Ludao is apparent in only a handful of MOTUs; therefore, we emphasize that individual species should be scrutinized prior to generalization to the community level. Nevertheless, connectivity over timescales exceeding this 18‐month study period cannot be ruled out, as long‐distance dispersal of migrants over ecological timescales (Noreen et al., 2009; van Oppen et al., 2008) or step‐wise transgenerational dispersal (Holstein et al., 2015; Vaz et al., 2016) may be sufficient to establish connectivity.

Pelagic larval duration (PLD) varies among coral species and positively correlates with dispersal distance (Shanks et al., 2003). Coral larvae are notoriously poor swimmers and exhibit limited ability to outmaneuver prevailing currents (Hata et al., 2017). Nevertheless, larval traits such as swimming behaviors, lipid content, energy availability, zooxanthellate acquisition, and buoyancy characteristics can profoundly impact dispersal potential (Harii et al., 2002; Richmond, 1987; Shanks, 2009; Szmant & Meadows, 2006). *Pocillopora damicornis* planulae are zooxanthellate and swim actively, enabling a long PLD of up to 212 days (Harrigan, 1972). In contrast, its sister species, *P. acuta*, is characterized by brief PLD, which leads to localized recruitment (Bahr et al., 2020). PLD is a critical trait for estimating connectivity and is available for many fish and other commercially important species; however, the PLDs of many coral species are still unknown. We hypothesize that extensive PLDs in *Pocillopora* may explain the widespread distribution of this genus in our survey. PLD could be a critical characteristic defining species which can disperse broadly and find refuge, and this possibility should be researched further. The prevalence of traits and/or behaviors which facilitate dispersal could explain the absence or low abundance of coral taxa restricted to one particular habitat in our experiment.

Reproductive modalities may also influence the dispersal of larvae tends to be associated with dispersal ability, although exceptions exist. Brooded larvae often settle soon after they are released which limits their ability to disperse (Nozawa & Harrison, 2005; Sakai, 1997; Warner et al., 2016) and short competency periods in brooded larvae may be responsible for producing localized dispersal patterns observed in high‐latitude communities (Tioho et al., 2001). In spawners, gametes may remain planktonic for several days, enhancing their potential for dispersal (Nozawa & Harrison, 2008). The larvae of spawners *A. millepora*, *A. tenuis*, and *M. digitata* possess a high lipid content which makes them buoyant, enabling dispersal by wind and currents and provides energy storage during periods of extended dispersal (Arai et al., 1993; Richmond, 1987); however, this trait is not present in all spawners. Nevertheless, brooders exhibiting high connectivity across vertical gradients (Hammerman et al., 2018; Serrano et al., 2016) and spawners possessing strong vertical genetic partitioning have been documented (Eckert et al., 2019; Serrano et al., 2014).

The S1 and S3 periods (April–October) spanned the main coral spawning period at Ludao (Dai & Fan, 1992; Nozawa et al., 2013). Most corals in Taiwan do not spawn during October–April, and observed patterns were consistent with expectations. *Pocillopora* dominance persisted during S1 and S3, but not during S2, when their abundance was comparable to other taxa. Kuo and Soong (2010) found variable pocilloporid recruitment during wet (May–September) and dry seasons (November to March) and observed similar patterns interannually. Fan et al. (2006) observed larval release in *Stylophora pistillata* and *Pocillopora damicornis* in Taiwan during winter months (February–March), providing the most likely explanation for the presence of these species. In Ludao, *P. damicornis* is uncommon while *P. verrucosa* is abundant (Y. Nozawa, personal communication). Recently, *P. verrucosa* has been found to brood in nearby Philippines (Villanueva et al., 2008) that wintertime *P. verrucosa* recruits could actually be brooded; however, further study is required. Several other pocilloporids can reproduce asexually, which could provide an alternative explanation for winter recruitment: *Pocillopora acuta* may generate planula asexually in the absence of sperm (Nakajima et al., 2018; Smith et al., 2019), and *P*. *damicornis* may produce clonal larvae, although this behavior may vary along latitudinal gradients (Miller & Ayre, 2004). Additionally, clonal reproduction in this species may be undertaken in response to disturbances (Sherman et al., 2006; Yeoh & Dai, 2010). Corals of the *Montipora* genus typically spawn between April and June in Taiwan, but may be subject to interannual variability (Lin & Nozawa, 2017); the small spat size (2.1mm) of the *Montipora* sp. (II) spat collected during S2 suggests it spawned late during this season, potentially explaining its retrieval during our winter survey. Lastly, it is possible that larvae with extended larval development released during the regular spawning season may remain viable to recruit during wintertime.

We emphasize the limitations in making inferences of the distribution of corals based on recruitment patterns; therefore, our conclusions warrant caution. Substrate choice is one of the most important factors determining the survival of coral larvae (Ritson‐Williams et al., 2009) and larvae may select locations that maximize their chances of surviving (Martinez & Abelson, 2013). We hypothesize that tile choice may influence results by providing a more favorable habitat to some while detracting others from settling. Indeed, Harriott and Fisk (1987) found the composition of acroporid and pocilloporid larvae settling on artificial substrates diverged from natural surfaces. Likewise, Burt et al. (2009) found recruitment densities varied among settlement substrate types. In some cases, diverging preferences may apply to close relatives: in azooxanthellate corals, *Tubastraea tagusensis* settles more densely on concrete substrates, while *Tubastraea coccinea* exhibits no such preference (Creed & De Paula, 2007). In addition, the date of initiation of the experiment could bias results toward MOTUs with later spawning dates if settlement tiles have not accumulated sufficient biofilm to promote metamorphosis (Webster et al., 2004). However, uncured terra‐cotta tiles do not have this effect on *Acropora millepora* larvae, indicating that some species are less selective than others (Heyward & Negri, 1999).

Fluorescent censusing enhanced our ability to find recruits of small size. The application of fluorescence as an aid for recruit censusing was partly successful, but we observed variable intensity within MOTUs and between depths. It is well documented that not all coral species are fluorescent (Alieva et al., 2008; Gruber et al., 2008; Kenkel et al., 2011; Roth et al., 2015); however, a thorough registry of fluorescent corals does not exist. Additionally, in fluorescent types, intraspecific variation may occur (Eyal et al., 2015; Wangpraseurt et al., 2019). We were unable to quantitatively measure variation in fluorescence in our recruits, but we hypothesize that variation across shallow and deep light environments, in combination with differences in light exposure due to settlement location (i.e., vertical/horizontally oriented tile and top/bottom of the tile) could induce variation. The intensity of fluorescence may be influenced by light climates, such as depth (Scucchia et al., 2020) and shading surrounding the coral (Lesser & Gorbunov, 2001; Ralph et al., 2002). Eyal et al. (2015) showed that fluorescent signals in some species are completely independent of light exposure, while in others, fluorescence may be lost in dark environments. Alternately, fluorescent signals may be impacted by coral health (Wangpraseurt, Larkum, et al., 2019) and dimmed fluorescent responses may indicate stress (Roth et al., 2015). Further study into interspecific and bathymetric variation in coral fluorescence during early‐life stages is warranted.

Our AUR design resulted in a simple, affordable, and convenient tool to study recruitment. Tiles can be fixed to the block in advance and lowered into place by divers, forgoing drilling underwater to fix recruitment tiles directly to the substrate. This is advantageous in mesophotic settings where bottom time is limited and fieldwork is expensive; however, carrying heavy blocks at depth can be physically taxing on divers. Because the design is modular, scaling the quantity of blocks and tiles to tailor this method to individual requirements is feasible. This study fulfills a need for information on mesophotic recruitment and how it contrasts with shallow reefs. These insights into local coral recruitment processes highlight the importance of early‐life stage dynamics on mesophotic coral reef demographics. In few MOTUs recruiting abundantly across a wide depth distribution, our results are consistent with the mesophotic depth acting as a refuge for shallow‐water communities. However, based on the evidence currently available, this tenet does not apply to most MOTUs which recruit scarcely and within their endemic depth range, warranting caution on generalizing this hypothesis at the community level. Nevertheless, connectivity over long timescales cannot be discredited and warrants further examination. More research is required to further expand our knowledge of recruitment and connectivity at depth, while delving into the physiological and environmental processes which affect them. As higher resolution molecular markers are developed, the resolution of molecular taxonomy will improve accordingly. Still, the present work represents a noteworthy improvement over traditional recruit identification. Future studies should strive to explore understudied geographical areas while developing innovative ways to overcome the challenges of surveying recruitment at depth.

## CONFLICTS OF INTEREST

The authors have no conflicts of interest to declare.

## AUTHOR CONTRIBUTION

**Derek Soto:** Conceptualization (equal); Data curation (equal); Formal analysis (equal); Funding acquisition (equal); Investigation (equal); Methodology (equal); Resources (equal); Software (equal); Validation (equal); Visualization (equal); Writing‐original draft (equal); Writing‐review & editing (equal). **Stéphane De Palmas:** Conceptualization (equal); Data curation (equal); Formal analysis (equal); Funding acquisition (equal); Investigation (equal); Methodology (equal); Validation (equal); Writing‐original draft (equal); Writing‐review & editing (equal). **Vianney Denis:** Conceptualization (equal); Formal analysis (equal); Investigation (equal); Methodology (equal); Validation (equal); Writing‐original draft (equal); Writing‐review & editing (equal). **Ming‐Jay Ho:** Conceptualization (equal); Investigation (equal); Methodology (equal); Resources. **Chaolun Allen Chen:** Conceptualization (equal); Formal analysis (equal); Funding acquisition (equal); Investigation (equal); Methodology (equal); Project administration (equal); Resources; Software; Supervision (equal); Writing‐original draft (equal); Writing‐review & editing (equal).

## PERMITS

Coral tissue samples were collected under Taitung County Government permit number 1040000285.

## 5 DATA ACCESSIBILITY

Coral recruit metadata and DNA sequences are publicly accessible on Dryad: https://doi.org/10.5061/dryad.msbcc2fz4.
